# Development of a prognostic signature of patients with esophagus adenocarcinoma by using immune-related genes

**DOI:** 10.1186/s12859-021-04456-2

**Published:** 2021-11-01

**Authors:** Xiangxin Zhang, Liu Yang, Ming Kong, Jian Ma, Yutao Wei

**Affiliations:** 1 Shihezi University School of Medicine, Shihezi, Xinjiang China; 2 Department of Thoracic Surgery, Shandong Second Provincial General Hospital, Shandong ENT Hospital, Jinan, Shandong China; 3 Shandong Cancer Institute (Shandong Cancer Hospital), Shandong First Medical University, Shandong Academy of Medical Sciences, Jinan, Shandong China; 4 Department of Thoracic Surgery, Jining First People’s Hospital, Jining, Shandong China

**Keywords:** Esophagus adenocarcinoma (EAC), Immune-related genes (IRGs), Prognostic signature, TCGA database, Cox regression analysis

## Abstract

**Background:**

Esophageal adenocarcinoma (EAC) is an aggressive malignancy with a poor prognosis. The immune-related genes (IRGs) are crucial to immunocytes tumor infiltration. This study aimed to construct a IRG-related prediction signature in EAC.

**Methods:**

The related data of EAC patients and IRGs were obtained from the TCGA and ImmPort database, respectively. The cox regression analysis constructed the prediction signature and explored the transcription factors regulatory network through the Cistrome database. TIMER database and CIBERSORT analytical tool were utilized to explore the immunocytes infiltration analysis.

**Results:**

The prediction signature with 12 IRGs (ADRM1, CXCL1, SEMG1, CCL26, CCL24, AREG, IL23A, UCN2, FGFR4, IL17RB, TNFRSF11A, and TNFRSF21) was constructed. Overall survival (OS) curves indicate that the survival rate of the high-risk group is significantly shorter than the low-risk group (*P* = 7.26e−07), and the AUC of 1-, 3- and 5- year survival prediction rates is 0.871, 0.924, and 0.961, respectively. Compared with traditional features, the ROC curve of the risk score in the EAC patients (0.967) is significant than T (0.57), N (0.738), M (0.568), and Stage (0.768). Moreover, multivariate Cox analysis and Nomogram of risk score are indicated that the 1-year and 3-year survival rates of patients are accurate by the combined analysis of the risk score, Sex, M stage, and Stage (The AUC of 1- and 3-years are 0.911, and 0.853).

**Conclusion:**

The 12 prognosis-related IRGs might be promising therapeutic targets for EAC.

**Supplementary Information:**

The online version contains supplementary material available at 10.1186/s12859-021-04456-2.

## Introduction

Esophageal cancer (EC) is an aggressive upper gastrointestinal malignancy and is ranked ten common malignancies worldwide [1]. EC is classified into two major histotypes: esophageal squamous cell carcinoma (ESCC) and esophageal adenocarcinoma (EAC). The incidence of EAC has increased significantly in recent years. Although artificial intervention and treatment of patients with high-risk factors and long-term exposure greatly influence the progress of EAC, whereas, it still has a poor prognosis rate with a 5-year survival rate of less than 20% [2]. Therefore, it is necessary to identify more diagnostic and prognostic biomarkers and effective therapeutic targets for EAC patients.

As we all know, the tumor microenvironment plays a vital role in cancer initiation and progression and in response to cancer treatment [3, 4]. As an indispensable part of the microenvironment, tumor-infiltrating immune cells are closely associated with the growth, invasion, and metastasis of carcinomas [5, 6]. In recent years, immunotherapy has been included in the multiple treatment guidelines of cancers and has become part of the standard treatment plan for numerous solid tumors [7–9]. For example, the immune checkpoint therapy targeting programmed death protein (PD-1) and programmed death-ligand 1 (PD-L1) have achieved encouraging results in the treatment of melanoma [10], advanced non-small cell lung cancer (NSCLC) [11, 12], and gastric cancer [13]. Nevertheless, not every patient has a good response to the currently recommended immunotherapy. The potential prognostic value of immune responses involving different cells may vary depending on the immune-related genes (IRGs) [14].

Recently, prognostic signatures based on IRGs have been constructed in a variety of tumors to develop individualized immune characteristics and improve the prognostic evaluation in immunotherapy, such as non-squamous non-small cell lung cancer [15], glioma [16], and hepatocellular carcinoma [17]. Although the previous studies have explored the predictive value of IRGs in the EC or EAC, the accuracy of each signature is different, and the IRGs included are difference [18–20]. Therefore, are there other differentially expressed immune-related genes (DEIRGs) that affect the prognosis of EAC patients? Compared with traditional features, how accurate is the prognosis prediction signature based on these DEIRGs? Is the signature based on EAC patients also applicable to ESCC patients? What transcription factors have potential regulatory relationships with DEIRGs? What are the important biological functions of these DEIRGs, and what signal transduction pathways do they participate in? What are the relationships between the expression level of DEIRGs in the prognostic signature, the abundance of tumor-infiltrating immune cells, and the frequency of copy number variation? These are the questions that our research needs to explore.

## Material and methods

### Data sources

The RNA-seq FPKM data of EAC and the corresponding clinical information were downloaded from the TCGA database, which included 80 EAC tissues and 10 normal esophagus tissues (Table 1). A total of 2,498 immune-related genes (IRGs) related to human cancers were obtained from the ImmPort database (https://www.immport.org/home) [21]. The transcription factor (TF) was acquired from the Cistrome database (https://www.cistrome.org/) [22]. To further assess the prognostic power of this signature, the transcriptome data of GSE72873 were downloaded from the Gene Expression Omnibus (GEO, https://www.ncbi.nlm.nih.gov/geo/), which included 48 EAC tissues. The related clinical data were obtained from the Supplemental data submitted by Krause et al. [23].

**Table 1:** The clinical characteristics of patients with EAC in TCGA

Clinical characteristics	Patients (n = 80)	Percentage (%)	Clinical characteristics	Patients (n = 80)	Percentage (%)
**Age**			**New events**		
≤ 65	36	45	NO	45	56.25
>65	44	55	YES	35	43.75
**Gender**			**Alcohol history**		
Female	11	13.75	NO	27	33.75
Male	69	86.25	YES	53	66.25
**Vital Statue**			**Barretts_esophagus**		
Alive	41	51.25	NO	48	60
Dead	39	48.75	YES	26	32.5
**Stage**			Unknow	6	7.5
Stage I	10	12.5	**neoplasm_histologic** **_grade**		
Stage II	26	32.5	G1	1	1.25
Stage III	34	42.5	G2	28	35
Stage IV	10	12.5	G3	25	31.25
**T**			GX	26	32.5
T1 + T2	30	37.5	**Chemotherapy**		
T3 + T4	48	60	NO	71	88.75
Tx	2	2.5	YES	9	11.25
**N**			**Radiation_therapy**		
N0	22	27.5	NO	60	75
N1-N3	55	68.75	YES	7	8.75
Nx	3	3.75	Unknow	13	16.25
**M**					
M0	70	87.5			
M1	10	12.5			

### Screening of differentially expressed IRGs, and differentially expressed TFs

According to the gene annotations from the GENCODE project (https://www.gencodegenes.org/) [24], the RNA-seq data of EAC was classified into lncRNA protein-encoding gene profile data. Differentially expressed genes (DEGs) between the tumor and normal tissue samples were identified from the protein-coding gene profile data by using the "limma" [25] package. The *P* < 0.05 and |log2 FC|> 1 were considered meaningful. The differentially expressed immune-related genes (DEIRGs) were further extracted from the list of DEGs.

### Functional enrichment analysis

Further exploring these DEIRGs may be involved in the Gene Ontology (GO) and the Kyoto Encyclopedia of Genes and Genomes (KEGG) pathways. The "cluster profile" [26] and" org.Hs.eg.db" packages were utilized to perform GO and KEGG analysis. The *P* < 0.01 was considered statistically significant.

### Prognostic model construction and evaluation

To further confirm the potential prognostic value of DEIRGs, univariate Cox regression analysis was used to assess the association between DEIRGs and survival data (*P* < 0.05). DEIRGs with statistical significance were then selected into the multivariate Cox regression analysis to establish a robust prognostic signature and calculate the risk score. The risk score calculation is based as follows = ∑regression coefficient (genie) × expression value (genie). According to the median risk score of the DEIRGs predictive model, the patients of TCGA and GSE72873 datasets were classified into high-risk and low-risk groups to assess the evaluation performance of this signature. The predictive performance of this signature was also assessed in ESCC patients based on the TCGA database. Besides, the receiver operating characteristic (ROC) curve and area under the ROC curve (AUC value) were performed to evaluate the prognostic model's predictive value. Moreover, to assess whether the risk score of prognostic-related DEIRGs can be a prognostic indicator independent of clinicopathological factors, univariate and multiple Cox regression analyses were utilized to evaluate the relationship between clinical data and risk score.

### Construction of nomogram and TF-mediated regulatory networks

According to the result of previous multivariate cox regression analysis, we further utilized the “survival” and “rms” packages to construct a nomogram, which can assist in the clinical interpretation and predict the survival probability of EAC patients. Moreover, calculated the time-dependent ROC curves and the AUC values of this Nomogram. Finally, the difference between the predicted results of the nomogram and the actual results were drawn in the calibration curve.

The transcription initiation process of eukaryotes is very complicated and requires the assistance of a variety of protein factors. The transcription factor (TF) plays an essential role in initiation by forming a transcription initiation complex with RNA polymerase II. To explore which transcription factors regulate the transcription of prognostic-related DEIRGs, we performed pearson correlation analysis on both TFs and DEIRGs. The thresholds were set as follows:|correlation value|> 0.3 and *P* < 0.001.

### The analysis of immune infiltration and copy number variation in 12 DEIEGss

To further explore the relationships between the expression level of DEIRGs in the prognostic signature and the abundance of tumor infiltrated immune cells. Firstly, based on the CIBERSORT (https://cibersort.stanford.edu/) [27], we evaluated the difference in the proportion of 22 immune cell subtypes in each sample of the high-risk and low-risk group. TIMER (https://cistrome.shinyapps.io/timer/) [28] is an online database that can comprehensively analyze multiple tumors infiltrated immune cells, including B CD4 + T cells, CD8 + T cells, Neutrophils, Macrophages, and Dendritic cells. Furthermore, the TIMER was utilized to evaluate the relationship between DEIRGs expression and tumor infiltrated immune cells. Moreover, we further evaluated the frequency of copy number variation of 12 DEIRGs that constructed the signature. The related copy number variation data of EAC patients were obtained from the UCSC Xena database (http://xena.ucsc.edu/).

### Statistical analyses

All statistical analyses and related visualization were conducted to determine independent prognostic factors using the R package (R software version 3.6. 3), GraphPad Prism 8, and Perl (5.30.1) software.

## Result

### Identification of DEGs and DEIRGs

**Fig. 1:**
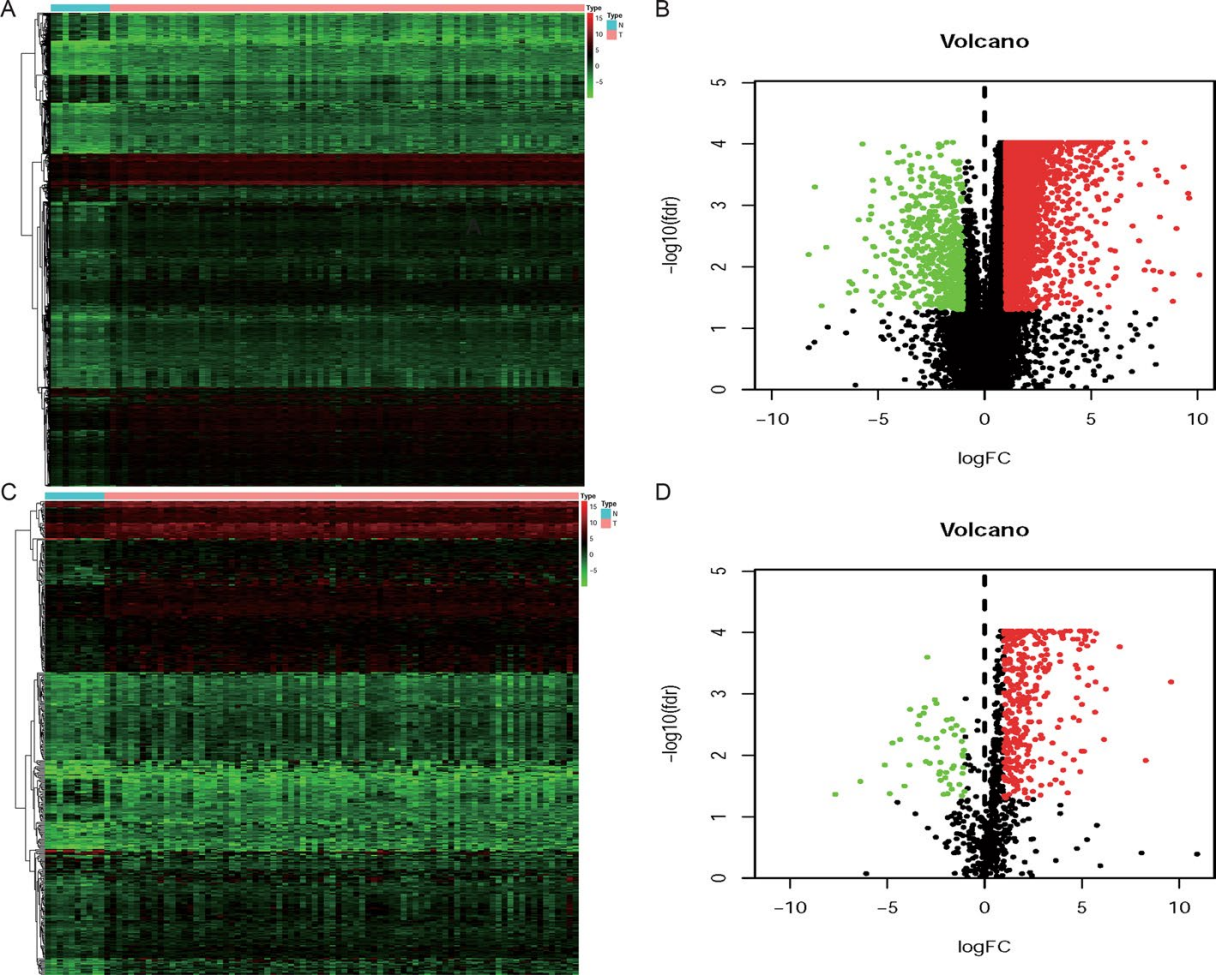
Differentially expressed genes (DEGs) between esophageal adenocarcinoma (EAC) and normal esophageal tissues. **a**, **b** The heatmap and volcano plot of DEGs in EAC and normal tissues. **c**, **d** The heatmap and volcano plot of differentially expressed Immune-related genes (DEIRGs) in EAC and normal tissues. The green spot represents the down-regulated gene, the red spot represents up-regulated gene

Based on the cut-off criteria of |log2 FC|> 1 and *P* < 0.05, a total of 3490 DEGs (2888 up-regulated and 602 down-regulated DEGs) were identified from the protein-encoding gene profile data between normal tissues and tumor tissues (Fig. 1a, b). Besides, according to the ImmPort database, a total of 399 DEIRGs (349 up- and 50 down-regulated DEIRGs) (Fig. 1c, d; Additional file 1: Table S1) were obtained from the list of DEGs.

### Functional enrichment analysis of DEIRGs

**Fig. 2:**
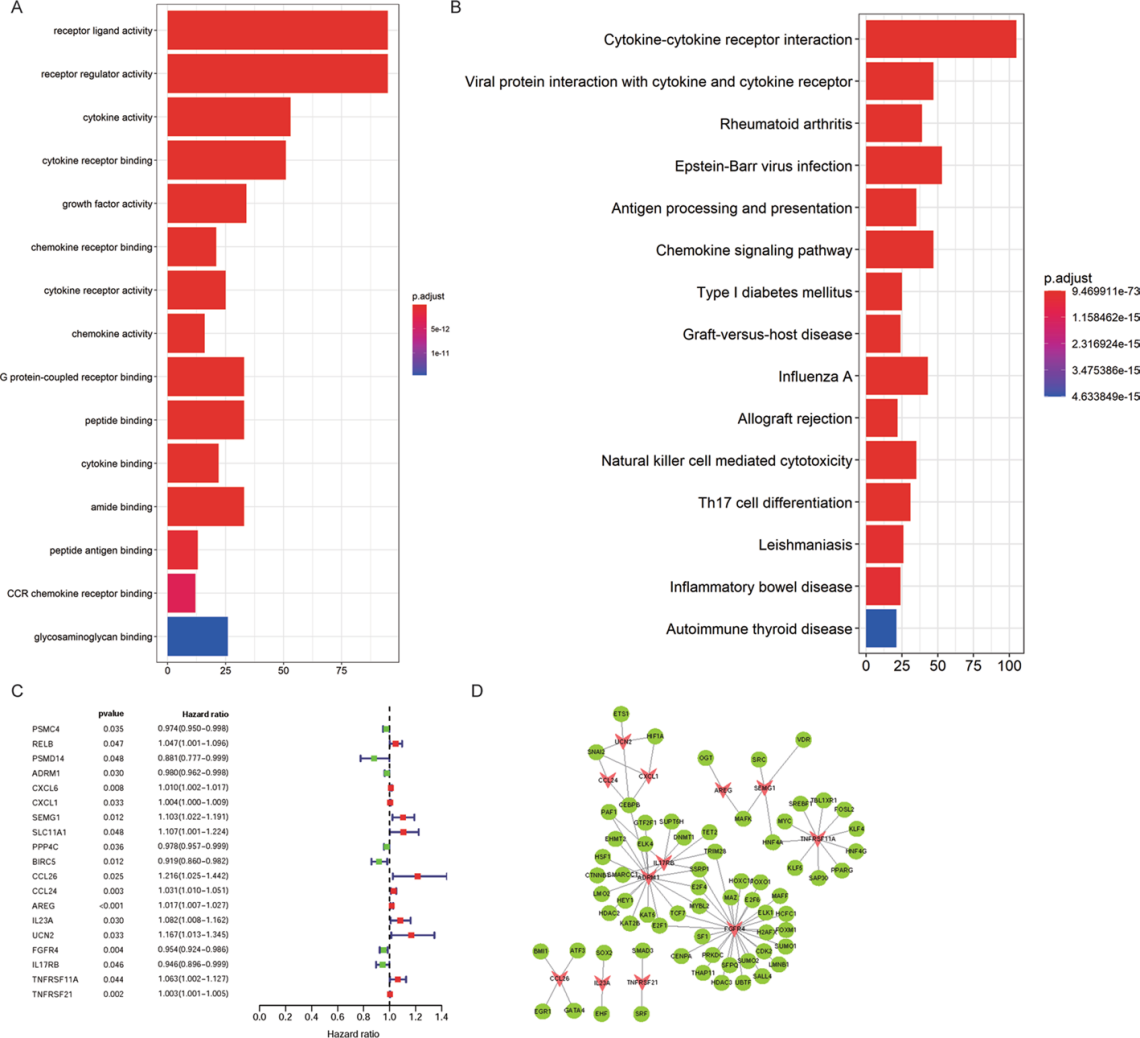
GO and KEGG pathway enrichment analyses. **a** Bar graph of GO enrichment analysis of the 399 DEIRGs. **b** Bar graph of KEGG pathway enrichment analysis of the 399 DEIRGs. **c** Based on Univariate Cox regression analysis obtained 19 prognostic DEIRGs. **d** The regulatory network consists of TFs (green circle) and 12 prognostic-related DEIRGs (red triangle). GO: Gene Ontology; KEGG: Kyoto Encyclopedia of Genes and Genomes

Under the criteria of *P* < 0.01, we have explored the biological characteristics of the DEIRGs. GO analysis indicates that these DEIRGs may be related to the biological characteristics of cell receptor-ligand activity, receptor regulator activity, cytokine activity, cytokine receptor binding, and growth factor activity (Fig. 2a). KEGG analysis shows that these DEIRGs are directly or indirectly involved in Cytokine-cytokine receptor interaction, Viral protein interaction with cytokine and cytokine receptor, and Antigen processing and presentation (Fig. 2b). Besides, DEIRGs are also involved in some vital signal pathways, such as the JAK-STAT signaling pathway, PI3K-Akt signaling pathway, PD-L1 expression, and PD-1 checkpoint pathway in the cancer MAPK signaling pathway (Additional file 2: Table S2).

### Construction and evaluation of IRG-related prognostic signature

**Fig. 3:**
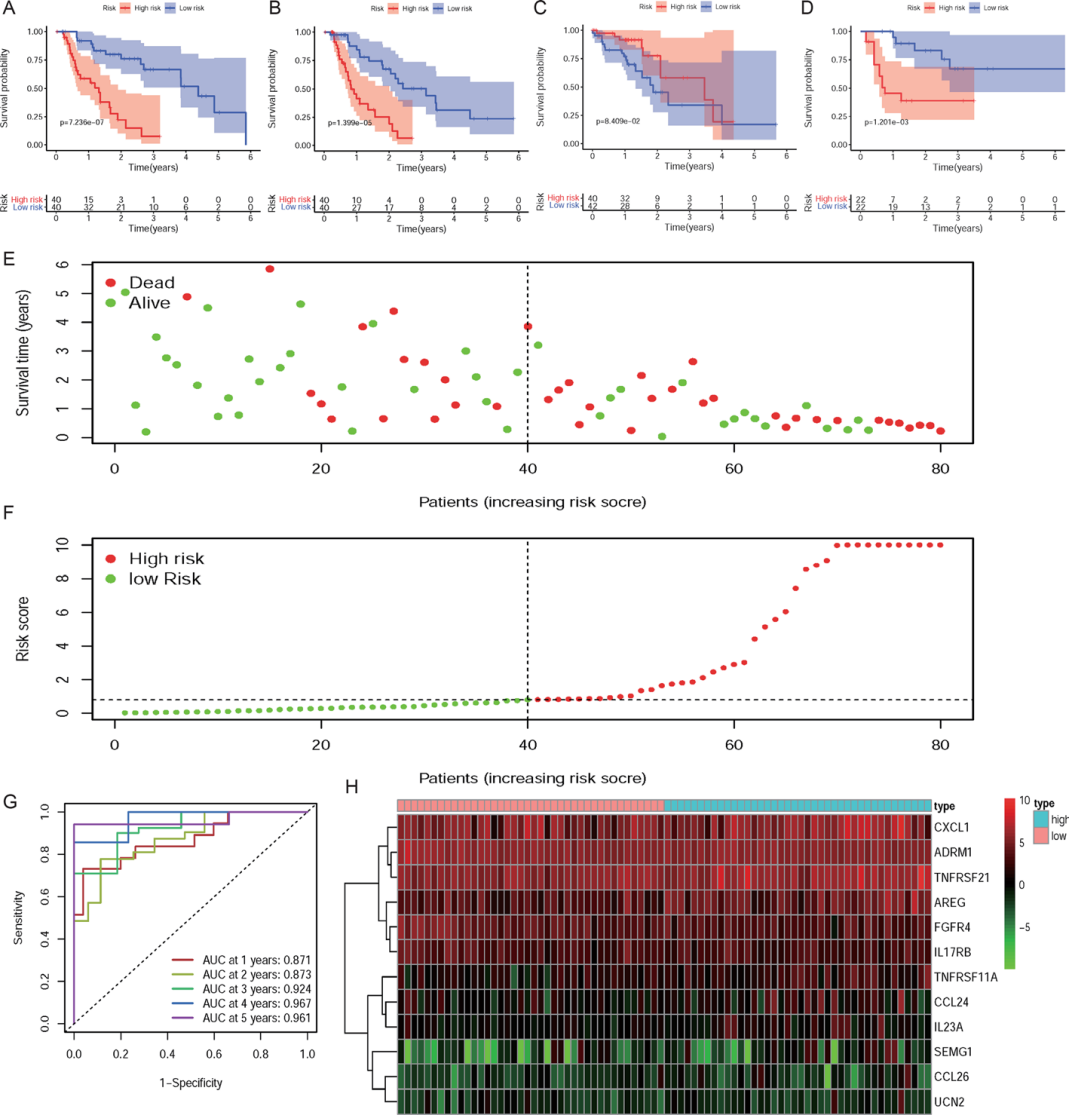
**a** The Kaplan–Meier survival analysis of overall survival between high- and low-risk groups of EAC patients; **b** The Kaplan–Meier survival analysis of disease-free survival between high- and low-risk groups of EAC patients; **c** The The Kaplan–Meier survival analysis of overall survival between high- and low-risk groups of ESCC patients; **d** The Kaplan–Meier survival analysis of overall survival between high- and low-risk groups of patients in GSE72873; **e** survival status of the patients in the low-risk group and high-risk group; **f** Ranking of the risk signature and distribution of the risk groups; **g** The receiver operating characteristic (ROC) curve of constructed signature in EAC; **h** The heatmap of expression profiles of the included genes

According to univariate cox regression analysis (*P* < 0.05), 19 prognostic-related DEIRGs have been selected (Fig. 2c). Subsequently, based on multivariate cox regression analysis, we finally obtained 12 DEIRGs to construct a prognostic signature (Table 2). The risk score of each EAC patient = (−0.01621*ADRM1) + (0.00499*CXCL1) + (0.06824*SEMG1) + (0.39269*CCL26) + (0.05053*CCL24) + (0.02883*AREG) + (0.11286*IL23A) + (−0.31860*UCN2) + (−0.03313*FGFR4) + (−0.03694*IL17RB) + (0.12972*TNFRSF11A) + (0.00438*TNFRSF21). According to the prognostic signature, EAC patients were divided into high-risk and low-risk groups. The overall survival (OS) of the EAC high-risk group is significantly shorter than that of the low-risk group (*P* = 7.26e-07) (Fig. 3a). The two-and-a-half-year survival rate of the high-risk group (0.0737, 95% CI 0.0124–0.439) is approximately about one-tenth that of the low-risk group (0.713, 95% CI 0.566–0.898) based on this prediction signature. Moreover, there are also significant differences in the disease-free survival (DFS) of patients in the high and low-risk groups (*P* = 1.399e−05) (Fig. 3b). The receiver operating characteristic (ROC) curves were used to evaluate the signature's predictive performance. The area under the ROC (AUC) curve of 1-, 3- and 5-year is 0.871, 0.924, and 0.961, respectively (Fig. 3g). However, the signature constructed by EAC patients' sequencing data is not suitable for ESCC patients (*P* = 8.409e − 02) (Fig. 3c). We determined its prognostic ability in the external test set GSE72873, and the result indicated that this signature could be used as a reliable predictor for OS in patients with EAC (*P* = 1.201e−03) (Fig. 3d). Besides, risk scores of all EAC patients were ranked to analyze the distributions of the prognostic-related DEIRGs. The distributions of survival status indicated that the high-risk group's survival rate and time were significantly decreased compared to the low-risk group (Fig. 3e, f). We have also shown the expression profiles of 12 prognostic-related DEIRGs in the high and low-risk groups (Fig. 3h).

**Table 2:** The list of 12 differentially expressed Immune-related genes (DEIRGs) in the prognostic prediction signature

Gene	Coef	HR	HR.95L	HR.95H	*P *value
ADRM1	−0.016206835	0.983923789	0.962921024	1.005384655	0.140978295
CXCL1	0.004991274	1.005003751	0.99888175	1.011163272	0.10936358
SEMG1	0.068236246	1.070618208	0.985792628	1.162742867	0.105188407
CCL26	0.392686523	1.480954071	1.188727511	1.845019098	0.000462561
CCL24	0.050527114	1.051825382	1.00424869	1.10165604	0.032396281
AREG	0.028832808	1.029252497	1.016310595	1.042359203	7.97E−06
IL23A	0.112855589	1.119470257	1.02553231	1.222012846	0.011610335
UCN2	−0.318601379	0.727165355	0.522352103	1.012285488	0.059077041
FGFR4	−0.03312774	0.967414974	0.932966514	1.003135394	0.073334515
IL17RB	−0.036938548	0.963735357	0.913151925	1.017120824	0.179325428
TNFRSF11A	0.129718312	1.138507634	1.06252719	1.219921378	0.000232274
TNFRSF21	0.004384263	1.004393888	1.002150781	1.006642016	0.000121351

### The prognostic-related DEIRGs and clinical features

**Fig. 4:**
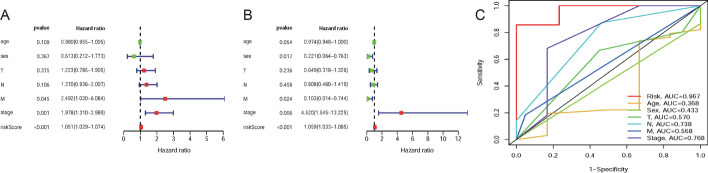
Clinical Value of the prognostic-related DEIRGs. The forest plots of EAC are based on **a** univariate Cox regression analysis and **b** multivariate Cox regression analysis; **c** The 4-year time-dependent ROC curve of risk score and traditional clinical features in the EAC patients

**Fig. 5:**
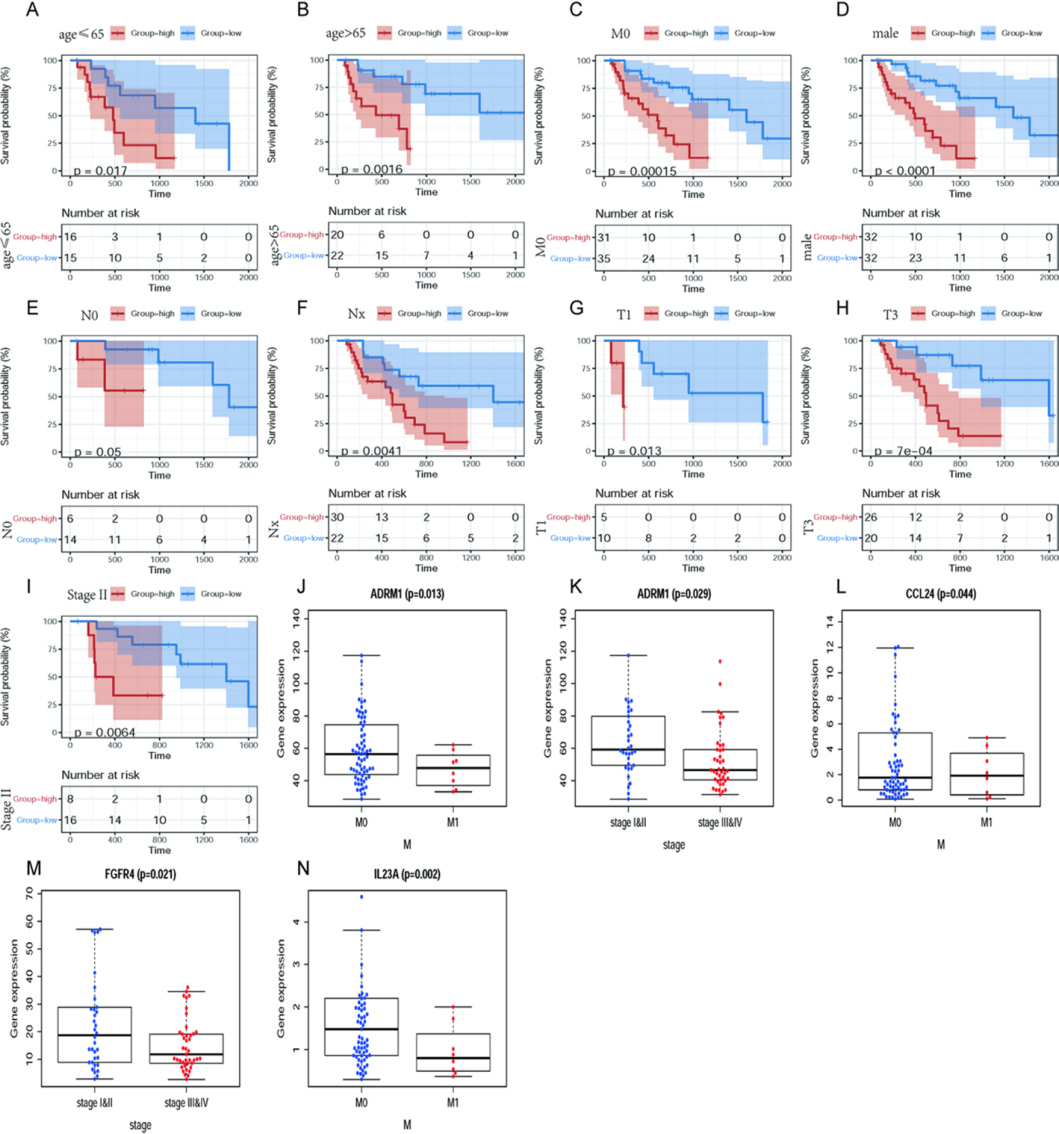
Stratification analysis of the association between the 12 prognostic-related DEIRGs and overall survival of EAC patients. **a** Ages ≤ 65 years; **b** ages > 65 years; **c** M0 stage; **d** male sex; **e** N0; **f** Nx; **g** T1 stage; **h** T3 stage; **i** stage II. Relationships between the expression of the 12 prognostic-related DEIRGs and clinicopathological factors in patients with EAC. **j** ADRM1 expression and M stage; **k** ADRM1 expression and stage; **l** CCL24 expression and M stage; **m** FGFR4 expression and stage; **n** IL23A expression and M stage

To testify whether the 12 prognostic-related DEIRGs could be utilized as the independent prognosis biomarkers of patients in EAC, we used univariate and multivariate cox regression analysis to evaluate the relationship between the clinical data and risk score. The univariate independent prognostic analysis shows that the M stage, Stage, and Risk score are significant prognostic factors (Fig. 4a, Table 3). However, multivariate independent prognosis analysis indicates that Sex, M stage, cancer stage, and risk score are significantly independent prognosis factors (Fig. 4b, Table 3). Compared with traditional features, the ROC curve of the risk score in the EAC patients (AUC = 0.967) is significant than T (AUC = 0.57), N (AUC = 0.738), M (AUC = 0.568), and cancer stage (AUC = 0.768) (Fig. 4c). Besides, the "beeswarm" package was used to evaluate the correlation between 12 DEIRGs and clinical characteristics of patients with EAC. Addtionally,we found that high-risk patients with certain characteristics have low survival times than low-risk groups (Fig. 5a–i). Moreover, in terms of the M stage, the median expression value of ADRM1 and IL23A in M0 is higher than that of M1, while the CCL24 in M0 is lower than M1. The median expression values of FGFR4 and ADRM1 in Stage I and II are higher than those in stage III-IV (Fig. 5j–n).

**Table 3:** Clinical characteristics and risk scores based on Univariate and multiple Cox regression analysis

Type	B	SE	z	HR	95% CI of HR	*P*-value
**Univariate Cox regression analysis**						
Age	−0.02058	0.012827	−1.60406	0.979635	0.955313–1.004576	0.108701
Sex	−0.48872	0.54149	−0.90255	0.613411	0.212243–1.772836	0.366767
T	0.200995	0.226366	0.88792	1.222619	0.784523–1.905346	0.374584
N	0.315018	0.19462	1.618634	1.370284	0.935723–2.006646	0.105526
M	0.912924	0.455467	2.004368	2.491597	1.020433–6.083766	**0.045031**
Stage	0.682306	0.210541	3.240722	1.978436	1.309503–2.989066	**0.001192**
RiskScore	0.050045	0.010946	4.572128	1.051319	1.029003–1.074116	**4.83E**−**06**
**Multiple Cox regression analysis**						
Age	−0.02668	0.013824	−1.93036	0.973668	0.947642–1.000409	0.053563
Sex	−1.50855	0.631636	−2.38833	0.22123	0.064142–0.762945	**0.016925**
T	−0.43244	0.364599	−1.18606	0.648926	0.317572–1.325997	0.235598
N	−0.21311	0.287253	−0.74189	0.808066	0.460182–1.418916	0.458151
M	−2.27121	1.008249	−2.25263	0.103187	0.014302–0.744473	**0.024283**
Stage	1.508601	0.54772	2.754327	4.520401	1.545102–13.22504	**0.005881**
RiskScore	0.056974	0.012569	4.532942	1.058629	1.032862–1.085031	**5.82E**−**06**

Bold inidcates clinical features are statistically signifcant in the Univariate- or Multivariate-cox regression analysis

### Nomogram and TF-mediated regulatory network of DEIRGs

**Fig. 6:**
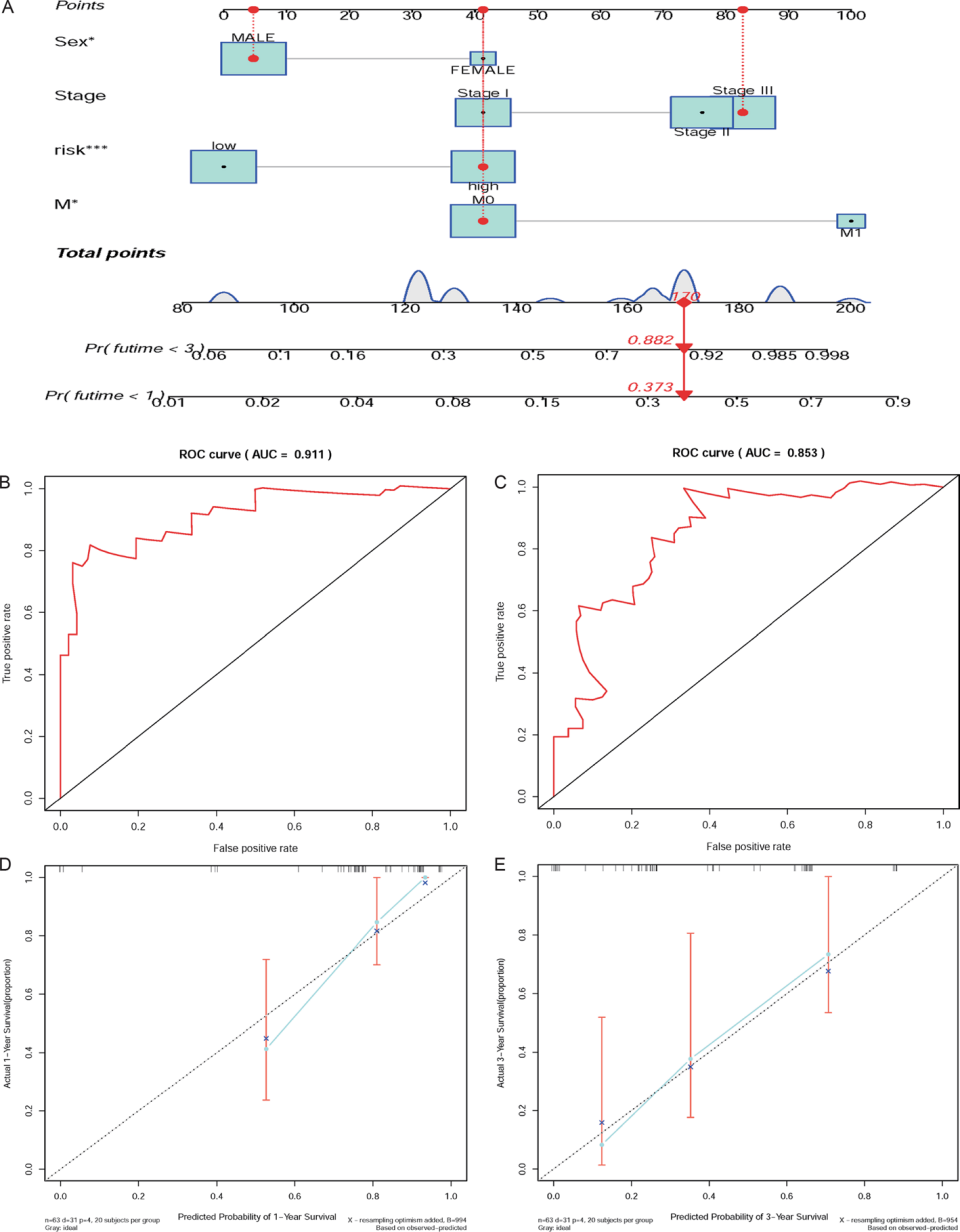
The performance evaluation and application of the nomogram. **a** Based on the total score, which was calculated by summing the scores of each item to calculated the survival rate of HCC patients for 1- and 3-year; **b**, **c** the ROC curves of nomogram in 1-and 3-year. The AUC of 1-and 3-year are 0.911 and 0.853 respectively; **d**, **e** The calibration curves of the nomogram in 1- and 3-year

Based on the result of multivariate cox regression analysis, M stage, grade, cancer stage, and the risk score were adopted into a nomogram which can assist in the clinical interpretation and be convenient to predict the survival rate of EAC patients. Based on the nomogram, the survival rate of 1- and 3-years can be assessed by summing the score of each item (Fig. 6a). The AUC of 1- and 3-years are 0.911, and 0.853 respectively (Fig. 6b,c). The calibration curves of the nomogram indicate that the predicted survival rates of 1- and 3- years have superior accuracy (Fig. 6d,e). Moreover, we performed a pearson correlation analysis on TFs and the 12 DEIRGs. Under the thresholds of |correlation value|> 0.3 and *P* < 0.001, a total of 85 TFs related to 12 DEIRGs were obtained (Fig. 2d; Additional file 3: Table S3). Among them, the top three DEIRGs with the most transcription factors are FGFR4 (27 related TFs), ADRM1 (20 related TFs), and TNFRSF11A (10 related TFs).

### Immunocyte infiltration analysis

**Fig. 7:**
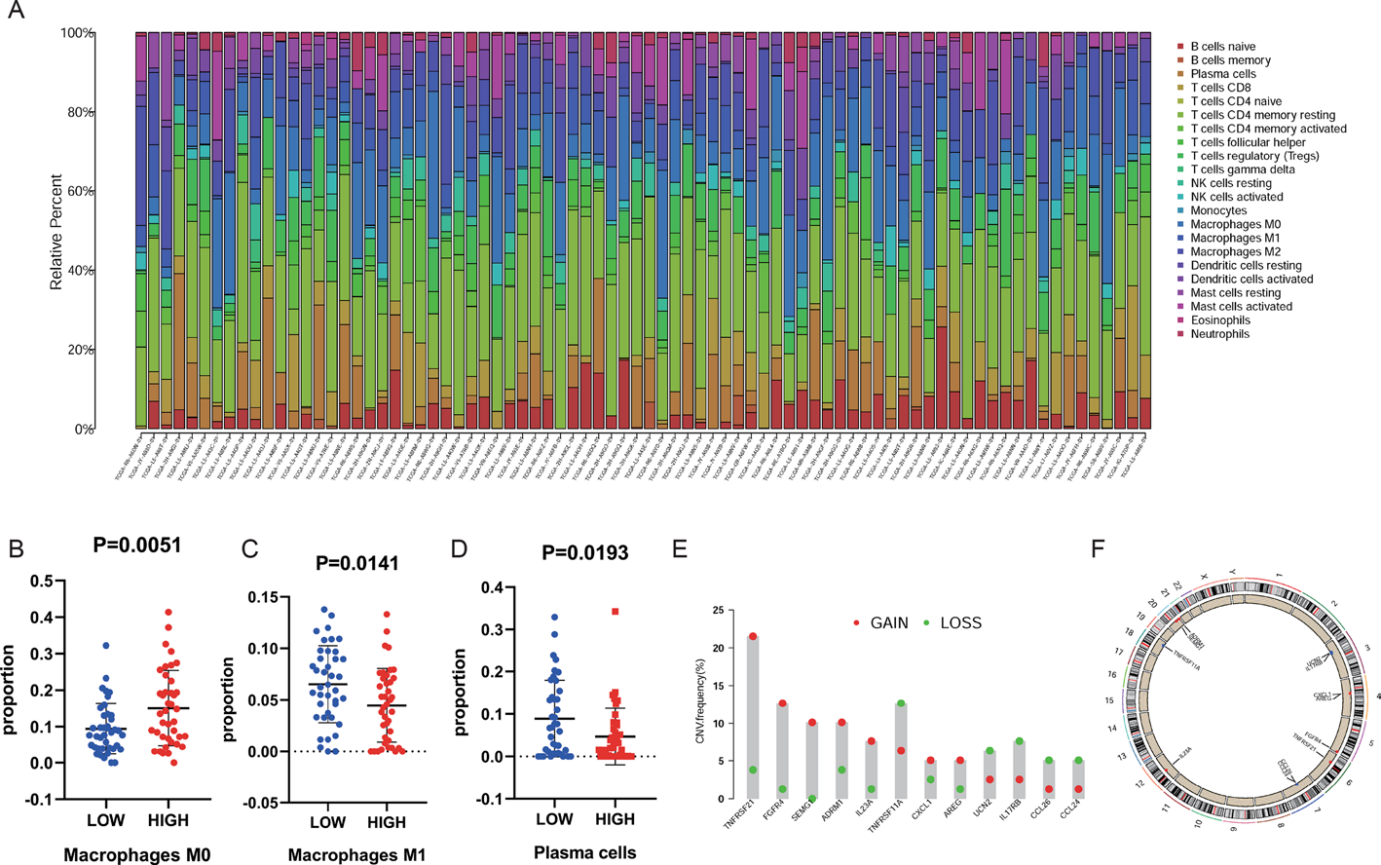
**a** The relative proportion of 22 immune cell subtypes in high- and low-risk EAC patients. **b**–**d** The proportions of M0, M1 and Plasma cells are different according to high and low risk groups; **e**, **f** The analysis of copy number variation in 12 DEIEGs

**Fig. 8:**
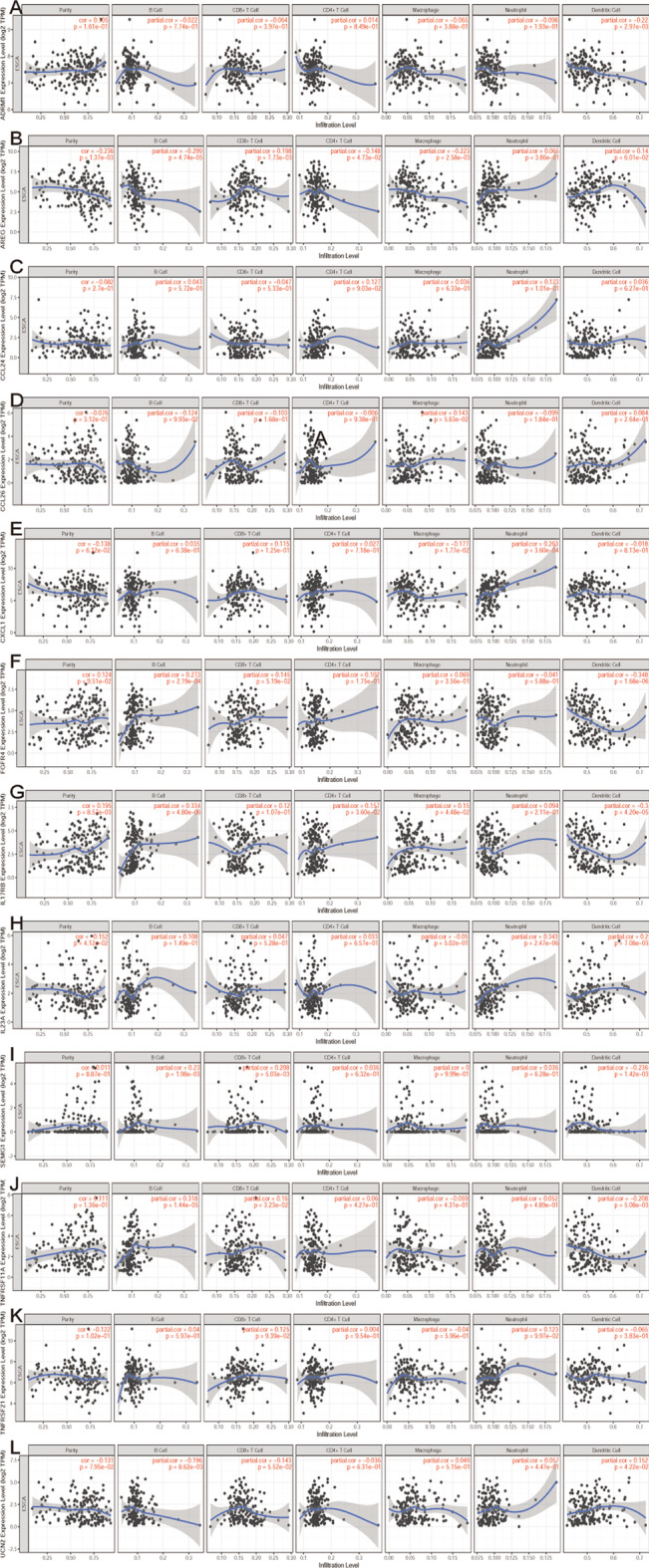
Infiltration level of 12 prognostic-related DEIRGs in 6 immune cells (B cells, CD4 + T cells, CD8 + T cells, dendritic cells, neutrophils, and macrophages). **a** ADRM1; **b** AREG; **c** CCL24; **d** CCL26; **e** CXCL1; **f** FGFR4; **g** L17RB; **h** IL23A; **i** SEMG1; **j** TNFRSF11A; **k** TNFRSF21; **l** UCN2

The CIBERSORT method was used to estimate differences in the infiltration of 22 immune cell types in the low-and high-risk group of EAC patients (Fig. 7a; Additional file 4: Table S4). The proportions of M0, M1, and Plasma cells are different according to high and low-risk groups (Fig. 7b–d). Moreover, the result indicated that most DEIRGs obtain copy number variation more frequently than lose copy number variation (Fig. 7e, f). Besides, the correlations between the 12 DEIRGs and 6 immune cell subtypes were investigated using TIMER (Fig. 8a–l). The results manifest that these prognostic-related DEIRGs have an immune infiltration relationship with at least one or more immune cells.

## Discussion

EC is one of the most aggressive malignant tumors globally. EAC is a histological subtype of EC with poor prognosis. Improvements in research and treatment indicate that the immune system and immune damage determine cancer occurrence and development [29, 30]. Accumulating evidence indicates that IRGs and the immune cellular microenvironment play a directly or indirectly pivotal role in carcinogenesis and tumor development [31, 32]. Our study analyzed the differential immune-related genes (DEIRGs) in EAC patients based on data from multiple public databases and constructed a robust predictive signature related to clinical prognosis to assess whether these DEIRGs could be the potential immune treatment targets of EAC.

A robust predictive signature was established based on integrated analysis of the consequence of univariate and multivariate cox regression analyses. Remarkably, the signature has an excellent ability to predict the specific survival rate of EAC (The AUC of 1- 3- and 5-year is 0.871, 0.924, and 0.961, respectively). The molecular mechanisms of DEIRGs show that it is functionally involved in cytokine activity, cytokine receptor binding, growth factor activity, and chemokine activity. At the same time, pathway analysis indicates that these DEIRGs are involved in the JAK-STAT signaling pathway, PI3K-Akt signaling pathway, PD-1 checkpoint pathway in cancer, and MAPK signaling pathway.

The comprehensive analysis of the predictive signature and clinical pathology data indicated that M stage, Stage, and risk scores can regard as independent prognostic factors in EAC. ADRM1 and FGFR4 were strongly correlated with tumor Stage, and ADRM1 was also associated with distant metastasis. The ADRM1-encoded protein, a member of the adhesion regulator 1 protein family, is a component of the proteasome. Studies have confirmed that the dysregulation of ADRM1-encoded protein in some malignancies is related to carcinogenesis, cell adhesion, and poor prognosis [33, 34]. Nevertheless, bis-benzylidine piperidone RA190, an ADRM1 Inhibitor, has the effect of reduced growth of multiple myeloma and ovarian cancer xenografts [35]. Equally, RA190 can also inhibit intrahepatic cholangiocarcinoma cell growth by inducing G2-M phase cell cycle arrest and nuclear factor κB (NF-κB)-regulated cell apoptosis [36]. Thus, in consideration of the important role of ADRM1 and its inhibitors in other tumors, more related research is worth exploring in EAC.

Fibroblast growth factor receptor 4 (FGFR4) is a member of a highly conserved tyrosine kinase family. Previous studies have indicated that various important pathways can be activated by FGFR4-related signaling, including Wnt/GSK-3β/β-catenin and the STAT signaling pathways [37, 38]. Additionally, FGFR4 drives tumor cell proliferation by inhibiting apoptosis induced by stress-related MST1/2 signaling [39]. Remarkably, compared with other FGFRs (FGFR1-3), FGFR4 with the unique cysteine residue within the ATP binding pocket (Cys552) [40]. Therefore, this makes it possible to develop some FGFR4-specific inhibitors to increase specificity and reduce the toxicity of FGFR targeted therapy. Moreover, some FGFR4 specific inhibitors (such as FGF401, BLU-554 and H3B-6527) combined with other treatments are also indispensable for improving clinical treatment effects [41–43]. FGF401 can act synergistically with vinorelbine to inhibit tumor growth and promote tumor apoptosis by inhibiting the FGF19/FGFR-4 signaling pathway [41].

As one of the IRGs that constructed signature, AREG and IL17RB were related to the carcinogenesis in some malignancies. The protein encoded by AREG is a member of the epidermal growth factor (EGF) family. As an EGFR ligand produced by Th9 cells, the study has shown that AREG can enhance Th9 cell differentiation [44]. Moreover, the AREG/EGFR signaling pathway may be involved in cancer progression through enhanced Treg cell function, leading to the activation of NFκB, which is closely related to neoadjuvant chemotherapy response and survival of patients with EAC [45]. In view of the key role of AREG in tumor immunity, it is worthy of further exploration in EAC.

IL17RB is plays a vital role in tumorigenesis. Amplified signaling of IL17RB and related ligand IL17B enhanced tumorigenicity in breast cancer cells and activated NF-κB to upregulate anti-apoptotic factor Bcl-2 and induced etoposide resistance [46]. Similary, the IL17B/IL17RB pathway promotes resistance to paclitaxel in breast tumors via the ERK1/2 pathway [47]. IL17RB activates multiple chemokine (such as CCL20/CXCL1/IL8/TFF1) expressions to enhance tumor cell invasion, macrophage, and endothelial cell recruitment at primary sites and cancer cell survival at distant organs [48]. However, targeting IL17B/IL17RB signaling with a newly derived anti–IL17RB antibody will block cancer cell metastasis and promote survival. Moreover, several studies have shown that the tumor microenvironment includes immune cells are critical players in tumor progression metastasis [49, 50]. Based on CIBERSORT analysis of the proportion of immune cells in the sample, indicate that compared with the low-risk group, the proportion of M0 in the high-risk group was higher, while M1 and plasma cells were lower, is worth exploring.

Although the previous studies have explored the predictive value of IRGs in the EC or EAC [18–20], the predicted signature we constructed (*P* = 7.26e−07, the AUC of 1-, 3- and 5-year is 0.871, 0.924 and 0.961 respectively) is more reliable than similar studies, and the prognostic ability of this signature is also assessed in the external test set GSE72874 (*P* = 1.201e−03). Moreover, compared with the study of Lan et al. [20], we focus more on which IRGs may participate and promote the occurrence of EAC. These IRGs and their current inhibitors may prepare for certain therapeutic interventions in patients with EAC.

Nevertheless, there are still certain limitations. The databases used in this study lack some important postoperative treatment information. Furthermore, the research is only conducted at the level of bioinformatics, and comprehensive in vitro and in vivo functional assays are needed further to explore the regulatory mechanism of DEIRGs in EAC.

## Conclusion

In general, the constructed prediction signature (ADRM1, CXCL1, SEMG1, CCL26, CCL24, AREG, IL23A, UCN2, FGFR4, IL17RB, TNFRSF11A, and TNFRSF21) based on IRGs is robust and promising. The DEIRGs in this signature can be used as potential biomarkers for EAC prognosis, which may help the development of individualized treatment.

## Supplementary Information

**Additional file 1**. **Supplement Table 1.** The list of 399 Differentially expressed immune-related genes (DEIRGs) including 349 up-regulated DEIRGs and 50 down-regulated DEIRGs.

**Additional file 2**. **Supplement Table 2.** Kyoto Encyclopedia of Genes and Genomes (KEGG) pathway analysis of 399 differentially expressed immune-related genes (DEIRGs).

**Additional file 3**. **Supplement Table 3.** Co-expression regulatory network of 85 transcription factors (TFs) and the 12 prognostic-related differentially expressed immune-related genes (DEIRGs).

**Additional file 4**. **Supplement Table 4.** The list of differences in the infiltration of 22 immune cell types in the low-and high-risk group of EAC patients.

## Data Availability

The datasets used and analyzed during the current study are available from The Cancer Genome Atlas (TCGA, https://portal.gdc.cancer.gov/), ImmPort database (ImmPort, https://www.immport.org/), and Cistrome database (https://www.cistrome.org/).
