# Supplementary material for: Development of a prognostic signature of patients with esophagus adenocarcinoma by using immune-related genes
Source: BMC Bioinformatics. 2021 Nov 1;22:536. doi: 10.1186/s12859-021-04456-2 (PMC8559413; doi:10.1186/s12859-021-04456-2)
Supplement: Supplementary file 1 — Additional file 1. Supplement Table 1. The list of 399 Differentially expressed immune-related genes (DEIRGs) including 349 up-regulated DEIRGs and 50 down-regulated DEIRGs. [file 12859_2021_4456_MOESM1_ESM.docx]

| **DEIRG** | **Gene name** |
| --- | --- |
| **Up-regulated** | *RASGRP3,ISG20L2,BCL10,GRB2,C3AR1,ENG,TXK,TBK1,ISG20,PSMD13,FAS,PSMC2,PGLYRP1,EIF2AK2,FGF19,*  *FURIN,CMTM2,PSMD11,IFNAR2,AEN,CMTM6,RARA,PTPN6,PPIA,CNTF,DDX58,PTAFR,IL27RA,SDC4,FABP2,*  *NFKBIB,PDGFB,TCF7L2,HCK,TNFRSF14,INPP5D,ADRBK1,HLA-E,HMGB1,PSMD14,OGFR,ADAR,NFKBIZ,*  *NFYA,CBLC,BMP8B,MX2,PPP4C,ABCC4,PROCR,TOR2A,CRLF3,C5AR1,TUBB3,PDGFRB,FPR1,IRF9,CDK4,*  *CMTM3,IRF3,SLC11A1,NMB,PML,CD14,PSME1,TAPBP,HDAC1,EDN3,PSMC4,PLXND1,CCL17,CYBB,RFX5,*  *TLR2,UCN2,VAV2,NPPA,IL12RB2,BCL3,VDR,UNC93B1,IFIH1,B2M,IL15RA,IL1A,RELB,ZC3HAV1L,SOCS1,IL17A,*  *IL7,TNFRSF1B,IKBKE,GMFG,CD72,SECTM1,NR6A1,HSP90AA1,OASL,NR1D1,CSPG5,TINAGL1,PGF,NR2C1,*  *TGFB2,HSP90AB1,TNFRSF10A,TNFSF13B,TNFRSF21,CACYBP,VEGFA,ADRM1,BID,UCN,CMTM1,PLSCR1,IL19,*  *CD4,LTA,CXCR6,APOBEC3F,TNFRSF11A,SHFM1,DLL4,CCL22,IL22RA1,CXCR4,TNFRSF10B,CD247,ROBO2,*  *TMSB10,HLA-DOB,APOBEC3G,PLXNC1,CIITA,CD28,CCR7,MX1HDGF,S100A5,IL2RA,PLXNA3,CCR5,LTB,NOX1,*  *CD3E,HSPA1B,EBI3,OXTR,NRAS,KRAS,FASLG,AREG,VCAM1,PIK3CG,VAV1,HLA-DPB1,FAM19A5,CETP,CST4*  *TNFAIP3,HLA-A,MSR1,CD70,TLR7,SEMA4G,LCN12,PSME2,HNF4G,APLNR,CD3D,PTGS2,BST2,TAP2,CD74,*  *TNFSF10,MET,INHBA,TNFRSF25,TNFRSF12A,LCP2,ITGB2,PSMB8,HLA-C,SEMA5B,CCL8,CTSB,PDCD1,PPBP,*  *TLR8,LYN,IRF1,S100A6,IRF7,STC2,RAC2,BMP8A,TYROBP,HNF4A,CHP2,IFI30,PSMD3,HLA-DMB,CTSS,KNG1,*  *TFRC,TYMP,HLA-DPA1,F2R,ICAM1,IL17RB,FCER1G,CSF2RA,IL2RB,FCGR3B,CD86,HLA-DOA,CCRL2,SAA2,*  *CCL4,CCR4,HLA-DMA,CSF3R,TNFSF9,STAT1,HLA-B,OAS1,SEMA7A,IL12RB1,CCL28,HLA-DRB1,HLA-F,CXCL5,*  *HLA-DRA,OXT,CXCL16,WFDC2,RSAD2,CCL26,HLA-DQA1,CMTM7,DMBT1,CXCR3,PF4,HLA-DQB1,LGR5,CKLF,IL21R,TNFRSF18,S100G,RARRES3,FABP6,TAP1,GZMB,NFKBIE,IFITM1,RBP4,CXCL13,GDF15,RLN2,TNFRSF4,*  *TNFRSF9,APLN,IL1B,CLDN4,CARD11,TNF,AMH,IL2RG,FGFR4,CXCL2,LIF,LCK,MUC4,FPR2,MDK,CCL25,*  *FCGR3A,BIRC5,NOX4,LEFTY1,GNLY,PLAUR,PROK2,C8G,LYZ,ULBP2,IFNG,PLAU,IL23A,ICOS,NR5A2,FGF4,*  *AQP9,CCL3,CTLA4,IL24,CCL3L3,IL32,R3HDML,OSM,CCL24,AGT,CCL4L2,ISG15,IL13RA2,HSPA6,CHIT1,PAEP,*  *MICB,SFTPA2,TNFSF11,MIA,PRKCG,MMP9,CXCL3,CXCL10,CXCL9,CCR8,CALCR,ULBP1,CCL15,LCN2,GAST,CCK,IL11,RBP2,TNFRSF11B,OLR1,RNASE2,CGB5,CCL7,IDO1,SAA1,NOS2,SPP1,SEMG1,CXCL11,CXCL1,CSF2,GUCCL20,REG3G,IL17C,ESM1,CXCL6,PF4V1,INHBE,MMP12,MTNR1A,CA2A,* |
| **Down-regulated** | *GKN1,VIP,ESRRG,PGC,ESRRB,PTGER3,PDIA2,VIPR2,DES,FIGF,CMA1,ANGPTL1,LIFR,GHR,LEPR,PTX3,*  *GREM2,NGFR,FABP4,CTSG,AR,MAPT,KL,SLIT2,OGN,SCTR,CXCL17,BMPR1B,NPY,AGTR1,GDF7,PDGFD,TGFBR3,CHGA,PTH1R,CCL21,SLC22,A17,IL33,DUOX1,CNTFR,NRG4,TGFB3,CCL14,RBP7,SYTL1,ADRB2,NPR3,SFTPD,NTF3,LTBP4* |

**Supplement table 1 .The list of 399 Differentially expressed immune-related genes (DEIRGs) including 349 up-regulated DEIRGs and 50 down-regulated DEIRGs.**
