# Supplementary material for: Development of a prognostic signature of patients with esophagus adenocarcinoma by using immune-related genes
Source: BMC Bioinformatics. 2021 Nov 1;22:536. doi: 10.1186/s12859-021-04456-2 (PMC8559413; doi:10.1186/s12859-021-04456-2)
Supplement: Supplementary file 2 — Additional file 2. Supplement Table 2. Kyoto Encyclopedia of Genes and Genomes (KEGG) pathway analysis of 399 differentially expressed immune-related genes (DEIRGs). [file 12859_2021_4456_MOESM2_ESM.docx]

**Supplement table 2. Kyoto Encyclopedia of Genes and Genomes (KEGG) pathway analysis of 399 differentially expressed immune-related genes (DEIRGs).**

| **ID** | **Description** | **P-value** | **Count** |
| --- | --- | --- | --- |
| hsa04060 | Cytokine-cytokine receptor interaction | 4.29E-75 | 105 |
| hsa04061 | Viral protein interaction with cytokine and cytokine receptor | 1.93E-39 | 47 |
| hsa05323 | Rheumatoid arthritis | 2.04E-30 | 39 |
| hsa05169 | Epstein-Barr virus infection | 2.93E-29 | 53 |
| hsa04612 | Antigen processing and presentation | 1.27E-28 | 35 |
| hsa04062 | Chemokine signaling pathway | 1.52E-24 | 47 |
| hsa04940 | Type I diabetes mellitus | 2.10E-24 | 25 |
| hsa05332 | Graft-versus-host disease | 3.23E-23 | 24 |
| hsa05164 | Influenza A | 5.31E-23 | 43 |
| hsa05330 | Allograft rejection | 1.52E-21 | 22 |
| hsa04650 | Natural killer cell mediated cytotoxicity | 8.46E-20 | 35 |
| hsa04659 | Th17 cell differentiation | 8.96E-19 | 31 |
| hsa05140 | Leishmaniasis | 8.95E-18 | 26 |
| hsa05321 | Inflammatory bowel disease | 1.55E-17 | 24 |
| hsa05320 | Autoimmune thyroid disease | 3.15E-16 | 21 |
| hsa05162 | Measles | 4.03E-16 | 32 |
| hsa04630 | JAK-STAT signaling pathway | 9.32E-16 | 34 |
| hsa05416 | Viral myocarditis | 6.09E-15 | 21 |
| hsa04658 | Th1 and Th2 cell differentiation | 1.24E-14 | 25 |
| hsa05167 | Kaposi sarcoma-associated herpesvirus infection | 3.81E-14 | 35 |
| hsa05152 | Tuberculosis | 1.55E-13 | 33 |
| hsa04657 | IL-17 signaling pathway | 1.95E-13 | 24 |
| hsa04672 | Intestinal immune network for IgA production | 2.10E-13 | 18 |
| hsa04064 | NF-kappa B signaling pathway | 2.69E-13 | 25 |
| hsa04145 | Phagosome | 2.80E-13 | 30 |
| hsa04640 | Hematopoietic cell lineage | 6.72E-13 | 24 |
| hsa05166 | Human T-cell leukemia virus 1 infection | 1.85E-12 | 35 |
| hsa04660 | T cell receptor signaling pathway | 2.14E-12 | 24 |
| hsa05145 | Toxoplasmosis | 1.17E-11 | 24 |
| hsa04514 | Cell adhesion molecules | 2.09E-10 | 26 |
| hsa04620 | Toll-like receptor signaling pathway | 7.61E-10 | 21 |
| hsa05310 | Asthma | 1.16E-09 | 12 |
| hsa05170 | Human immunodeficiency virus 1 infection | 1.65E-09 | 30 |
| hsa05163 | Human cytomegalovirus infection | 1.69E-09 | 31 |
| hsa04668 | TNF signaling pathway | 3.16E-09 | 21 |
| hsa05150 | Staphylococcus aureus infection | 7.11E-09 | 19 |
| hsa05160 | Hepatitis C | 1.64E-08 | 24 |
| hsa04380 | Osteoclast differentiation | 3.76E-08 | 21 |
| hsa04662 | B cell receptor signaling pathway | 1.39E-07 | 16 |
| hsa05143 | African trypanosomiasis | 1.39E-07 | 11 |
| hsa04933 | AGE-RAGE signaling pathway in diabetic complications | 4.55E-07 | 17 |
| hsa03050 | Proteasome | 1.56E-06 | 11 |
| hsa04217 | Necroptosis | 1.61E-06 | 21 |
| hsa05340 | Primary immunodeficiency | 1.83E-06 | 10 |
| hsa05235 | PD-L1 expression and PD-1 checkpoint pathway in cancer | 2.44E-06 | 15 |
| hsa04010 | MAPK signaling pathway | 2.68E-06 | 30 |
| hsa05146 | Amoebiasis | 3.01E-06 | 16 |
| hsa04625 | C-type lectin receptor signaling pathway | 3.91E-06 | 16 |
| hsa05168 | Herpes simplex virus 1 infection | 4.15E-06 | 42 |
| hsa04623 | Cytosolic DNA-sensing pathway | 6.80E-06 | 12 |
| hsa05161 | Hepatitis B | 8.15E-06 | 20 |
| hsa05322 | Systemic lupus erythematosus | 8.77E-06 | 18 |
| hsa05142 | Chagas disease | 1.38E-05 | 15 |
| hsa04664 | Fc epsilon RI signaling pathway | 1.55E-05 | 12 |
| hsa04080 | Neuroactive ligand-receptor interaction | 1.95E-05 | 31 |
| hsa05144 | Malaria | 2.57E-05 | 10 |
| hsa05165 | Human papillomavirus infection | 2.83E-05 | 30 |
| hsa04151 | PI3K-Akt signaling pathway | 4.04E-05 | 31 |
| hsa05133 | Pertussis | 4.92E-05 | 12 |
| hsa05134 | Legionellosis | 8.39E-05 | 10 |
| hsa04350 | TGF-beta signaling pathway | 9.94E-05 | 13 |
| hsa04015 | Rap1 signaling pathway | 0.00011798 | 21 |
| hsa04621 | NOD-like receptor signaling pathway | 0.000131364 | 19 |
| hsa04622 | RIG-I-like receptor signaling pathway | 0.000482538 | 10 |
| hsa05418 | Fluid shear stress and atherosclerosis | 0.000495336 | 15 |
| hsa05215 | Prostate cancer | 0.000526145 | 12 |
| hsa05203 | Viral carcinogenesis | 0.001572979 | 18 |
| hsa05205 | Proteoglycans in cancer | 0.001662008 | 18 |
| hsa04670 | Leukocyte transendothelial migration | 0.002201391 | 12 |
| hsa05218 | Melanoma | 0.002401844 | 9 |
| hsa04014 | Ras signaling pathway | 0.002782252 | 19 |
| hsa04210 | Apoptosis | 0.003457545 | 13 |
| hsa01521 | EGFR tyrosine kinase inhibitor resistance | 0.004533069 | 9 |
| hsa05221 | Acute myeloid leukemia | 0.005490868 | 8 |
| hsa05219 | Bladder cancer | 0.005799316 | 6 |
| hsa05211 | Renal cell carcinoma | 0.006564085 | 8 |
| hsa04917 | Prolactin signaling pathway | 0.007156851 | 8 |
| hsa04610 | Complement and coagulation cascades | 0.007337874 | 9 |
| hsa05210 | Colorectal cancer | 0.007911822 | 9 |
| hsa04614 | Renin-angiotensin system | 0.012835625 | 4 |
| hsa04666 | Fc gamma R-mediated phagocytosis | 0.012937907 | 9 |
| hsa04360 | Axon guidance | 0.015370045 | 14 |
| hsa04540 | Gap junction | 0.026093301 | 8 |
| hsa04923 | Regulation of lipolysis in adipocytes | 0.027307187 | 6 |
| hsa04810 | Regulation of actin cytoskeleton | 0.027612761 | 15 |
| hsa04024 | cAMP signaling pathway | 0.029676705 | 15 |
| hsa04550 | Signaling pathways regulating pluripotency of stem cells | 0.030838948 | 11 |
| hsa04370 | VEGF signaling pathway | 0.031715899 | 6 |
| hsa05214 | Glioma | 0.032020442 | 7 |
| hsa05212 | Pancreatic cancer | 0.034092187 | 7 |
| hsa05220 | Chronic myeloid leukemia | 0.034092187 | 7 |
| hsa04072 | Phospholipase D signaling pathway | 0.038238059 | 11 |
| hsa05135 | Yersinia infection | 0.038472298 | 10 |
| hsa04144 | Endocytosis | 0.04505414 | 16 |
| hsa05010 | Alzheimer disease | 0.045118783 | 22 |
| hsa05231 | Choline metabolism in cancer | 0.045299112 | 8 |
| hsa05202 | Transcriptional misregulation in cancer | 0.048743713 | 13 |
