# Supplementary material for: Development of a prognostic signature of patients with esophagus adenocarcinoma by using immune-related genes
Source: BMC Bioinformatics. 2021 Nov 1;22:536. doi: 10.1186/s12859-021-04456-2 (PMC8559413; doi:10.1186/s12859-021-04456-2)
Supplement: Supplementary file 3 — Additional file 3. Supplement Table 3. Co-expression regulatory network of 85 transcription factors (TFs) and the 12 prognostic-related differentially expressed immune-related genes (DEIRGs). [file 12859_2021_4456_MOESM3_ESM.docx]

**Supplement table 3 Co-expression regulatory network of 85 transcription factors (TFs) and the 12 prognostic-related differentially expressed immune-related genes (DEIRGs).**

| TF | IRG | Cor | P-value |
| --- | --- | --- | --- |
| PRKDC | FGFR4 | 0.400084 | 0.000236 |
| SUMO2 | FGFR4 | 0.558644 | 7.24E-08 |
| CENPA | FGFR4 | 0.451884 | 2.58E-05 |
| FOXO1 | FGFR4 | -0.41486 | 0.00013 |
| CDK2 | FGFR4 | 0.532872 | 3.61E-07 |
| SF1 | FGFR4 | 0.503317 | 1.95E-06 |
| HDAC3 | FGFR4 | 0.441063 | 4.22E-05 |
| E2F6 | FGFR4 | 0.430715 | 6.66E-05 |
| MYBL2 | FGFR4 | 0.371961 | 0.00068 |
| UBTF | FGFR4 | 0.412092 | 0.000146 |
| H2AFX | FGFR4 | 0.495275 | 3.00E-06 |
| FOXM1 | FGFR4 | 0.409029 | 0.000165 |
| SFPQ | FGFR4 | 0.375395 | 0.000601 |
| MAZ | FGFR4 | 0.516322 | 9.47E-07 |
| SUMO1 | FGFR4 | 0.451645 | 2.61E-05 |
| SALL4 | FGFR4 | 0.570748 | 3.25E-08 |
| LMNB1 | FGFR4 | 0.428024 | 7.48E-05 |
| MAFF | FGFR4 | -0.37644 | 0.000578 |
| HCFC1 | FGFR4 | 0.373673 | 0.00064 |
| E2F4 | FGFR4 | 0.403558 | 0.000206 |
| HOXC11 | FGFR4 | 0.536981 | 2.82E-07 |
| TRIM28 | FGFR4 | 0.567671 | 3.99E-08 |
| THAP11 | FGFR4 | 0.362327 | 0.000957 |
| SSRP1 | FGFR4 | 0.497896 | 2.61E-06 |
| ELK1 | FGFR4 | 0.397613 | 0.00026 |
| E2F1 | FGFR4 | 0.421187 | 0.0001 |
| TCF7 | FGFR4 | 0.442369 | 3.98E-05 |
| SNAI2 | UCN2 | 0.756989 | 4.54E-16 |
| CEBPB | UCN2 | 0.500569 | 2.26E-06 |
| ETS1 | UCN2 | 0.362615 | 0.000948 |
| HIF1A | UCN2 | 0.412111 | 0.000146 |
| SOX2 | IL23A | 0.368939 | 0.000758 |
| EHF | IL23A | 0.371977 | 0.00068 |
| SMARCC1 | ADRM1 | 0.372796 | 0.00066 |
| HDAC2 | ADRM1 | 0.37389 | 0.000635 |
| KAT5 | ADRM1 | 0.403522 | 0.000206 |
| MYBL2 | ADRM1 | 0.729573 | 1.65E-14 |
| KAT2B | ADRM1 | -0.36631 | 0.000832 |
| GTF2F1 | ADRM1 | 0.376882 | 0.000569 |
| HSF1 | ADRM1 | 0.440698 | 4.29E-05 |
| DNMT1 | ADRM1 | 0.446894 | 3.25E-05 |
| PAF1 | ADRM1 | 0.369457 | 0.000744 |
| E2F4 | ADRM1 | 0.423502 | 9.07E-05 |
| ELK4 | ADRM1 | -0.39384 | 0.000301 |
| TET2 | ADRM1 | -0.40898 | 0.000165 |
| SSRP1 | ADRM1 | 0.401908 | 0.00022 |
| CEBPB | ADRM1 | 0.370357 | 0.000721 |
| CTNNB1 | ADRM1 | 0.475191 | 8.43E-06 |
| EHMT2 | ADRM1 | 0.404945 | 0.000195 |
| LMO2 | ADRM1 | 0.482469 | 5.84E-06 |
| E2F1 | ADRM1 | 0.474422 | 8.76E-06 |
| HEY1 | ADRM1 | 0.446977 | 3.23E-05 |
| TCF7 | ADRM1 | 0.433835 | 5.81E-05 |
| SNAI2 | CXCL1 | 0.504764 | 1.80E-06 |
| CEBPB | CXCL1 | 0.449152 | 2.93E-05 |
| HIF1A | CXCL1 | 0.446011 | 3.38E-05 |
| MAFK | SEMG1 | 0.401028 | 0.000227 |
| VDR | SEMG1 | 0.38241 | 0.000464 |
| HNF4A | SEMG1 | 0.641696 | 1.42E-10 |
| SRC | SEMG1 | 0.390814 | 0.000338 |
| SMARCC1 | IL17RB | 0.370007 | 0.00073 |
| SUPT5H | IL17RB | 0.435686 | 5.36E-05 |
| PAF1 | IL17RB | 0.507405 | 1.56E-06 |
| TRIM28 | IL17RB | 0.367045 | 0.000811 |
| SSRP1 | IL17RB | 0.400569 | 0.000232 |
| EGR1 | CCL26 | 0.409406 | 0.000163 |
| BMI1 | CCL26 | 0.383607 | 0.000444 |
| ATF3 | CCL26 | 0.383481 | 0.000446 |
| GATA4 | CCL26 | 0.526721 | 5.20E-07 |
| SMAD3 | TNFRSF21 | 0.379782 | 0.000512 |
| SRF | TNFRSF21 | 0.657302 | 3.53E-11 |
| SNAI2 | CCL24 | 0.506507 | 1.64E-06 |
| CEBPB | CCL24 | 0.440792 | 4.27E-05 |
| MAFK | AREG | 0.445841 | 3.40E-05 |
| OGT | AREG | 0.361544 | 0.000984 |
| SAP30 | TNFRSF11A | 0.494043 | 3.21E-06 |
| KLF4 | TNFRSF11A | 0.389621 | 0.000354 |
| MYC | TNFRSF11A | 0.487606 | 4.49E-06 |
| HNF4G | TNFRSF11A | 0.461505 | 1.64E-05 |
| HNF4A | TNFRSF11A | 0.435554 | 5.39E-05 |
| PPARG | TNFRSF11A | 0.434137 | 5.74E-05 |
| SREBF1 | TNFRSF11A | 0.370239 | 0.000724 |
| KLF5 | TNFRSF11A | 0.385998 | 0.000406 |
| FOSL2 | TNFRSF11A | 0.380856 | 0.000492 |
| TBL1XR1 | TNFRSF11A | 0.426845 | 7.87E-05 |
